# Correlation between high hair cortisol level and intracranial aneurysm rupture

**DOI:** 10.1097/MD.0000000000026193

**Published:** 2021-06-04

**Authors:** You-Sub Kim, Sung-Pil Joo, Dong-Jun Song, Tae-kyu Lee, Tae-Sun Kim

**Affiliations:** Department of Neurosurgery, Chonnam National University Hospital and Medical School, Gwangju, Republic of Korea.

**Keywords:** aneurysmal rupture, chronic stress, hair cortisol

## Abstract

Measurement of cortisol in hair is a reliable method for determining long-term cortisol exposure reflecting chronic stress. Research using hair cortisol concentration has been limited to mainly cardiometabolic diseases. The association between hair cortisol concentration and aneurysmal rupture has not yet been studied. We aimed to investigate the relationship between the degree of chronic stress as measured by hair cortisol concentration and aneurysmal rupture.

Sixty-eight patients diagnosed with intracranial aneurysms were included in this study (ruptured group, 30; unruptured group, 38). Hair cortisol was measured in 3-cm hair segments, reflecting roughly 3 months of hair growth. For a risk factor analysis, patient-specific factors and aneurysm-specific factors as well as hair cortisol concentration were investigated.

Hair cortisol concentrations were significantly higher in the ruptured group than in the unruptured group (55.8 ± 22.0 ng/dL vs. 19.1 ± 6.4 ng/dL; *P* < .001). High hair cortisol concentration was found to be an independent risk factor for aneurysmal rupture (odds ratio [OR]: 2.245, 95% confidence interval [CI]: 1.825–2.753; *P* = .013). Additionally, a history of cerebrovascular disease was significantly associated with an increased risk of aneurysmal rupture (OR: 1.577, 95% CI: 1.099–2.262; *P* = .040).

Based on our results, we suggest that chronic stress as measured by hair cortisol concentration could be an independent risk factor for intracranial aneurysmal rupture.

## 1 Introduction

Unruptured intracranial aneurysms (UIAs) are somewhat common, found in approximately 3% of the adult population worldwide.^[1,2]^ Although the rupture rates of UIAs vary across countries, most studies have found an annual risk of rupture approximating 1%.^[3]^ Despite the relatively small number of rupture events, epidemiologic studies demonstrate that 60% of patients with subarachnoid hemorrhage from aneurysmal rupture either died or became severely disabled.^[4]^ Neurosurgeons are often faced with the dilemma of whether to treat unruptured aneurysms or to manage them conservatively. What is clear is that a combination of modifiable risk factor prevention and management may be necessary to reduce the risk of aneurysmal rupture. However, modifiable risk factors are mainly limited to cigarette smoking, hypertension, and alcohol consumption.^[5,6]^ Moreover, these well-known risk factors have inconsistent relationships to aneurysmal rupture and are consequently controversial.^[2]^

Efforts have been made to find other modifiable risk factors and there has been growing evidence that chronic psychological stress is significantly associated with cerebrovascular health including ischemic stroke or aneurysmal rupture.^[7–9]^ However, questionnaires were used to measure stress in previous studies, which posed a risk of recall bias and did not reflect the biologic status.

Chronic stress can have significant impact on the endocrine system due to sustained high levels of the chemicals released in the “fight or flight” response. In particular, cortisol, which is synthesized from cholesterol, is secreted in response to biochemical stress via suppression of the hypothalamic–pituitary–adrenal axis. Previous studies have shown that chronic stress is associated with an elevated cortisol level.^[10,11]^ Long-term elevated cortisol levels are related to the majority of negative clinical and health disorders including coronary heart disease and cerebrovascular disease.^[12–14]^ This suggests that long-term elevated cortisol levels might have a significant impact on vascular health.

In general, cortisol in biological fluids including serum, saliva, and urine is secreted in a circadian rhythm.^[15]^ Therefore, the timing of sample testing is crucial when measuring cortisol in biological fluids. In addition, a single measurement of cortisol in biological fluids is affected by acute stress, and therefore, poorly reflects long-term cortisol levels.

In contrast to biological fluids, the measurement of cortisol in hair, with a relatively constant growth rate of approximately 1 cm/month, can provide biological information related to long-term cortisol level revealing cortisol exposure over a one-month period and is therefore not influenced by the time of sample collection and acute stress.^[10,16,17]^ Previous studies have found that high hair cortisol concentration is associated with chronic stress.^[11,18]^ Therefore, the measurement of hair cortisol concentration could be a more reliable tool to study the association between chronic stress and aneurysmal rupture.

Research using hair cortisol concentration has been limited in that it has focused on only a few diseases, mainly those related to cardiometabolic health.^[19–21]^ The association between hair cortisol concentration and aneurysmal rupture has not yet been studied.

In the present study, we investigated whether there is an association between aneurysmal rupture and chronic stress as measured by hair cortisol concentration.

## 2 Methods

### 2.1 Study population and data collection

This study was approved by the institutional review board of Chonnam National University Hospital, Republic of Korea. Informed consent was obtained from the patients or from their families. Sixty-eight randomly selected patients who were diagnosed with intracranial aneurysms between July 2015 and August 2016 were included in the study. Patients were divided into two groups: a ruptured group (study group, 30 patients) and an unruptured group (control group, 38 patients). To assess the risk factors for aneurysmal rupture, baseline risk factors and hair cortisol levels were analyzed. Patient-specific factors included age, sex, body mass index (BMI), family history, smoking status, and other underlying chronic diseases.

Presence of hypertension (HTN), diabetes mellitus (DM), dyslipidemia, a history of coronary artery disease (CAD) including myocardial infarction or angina pectoris, and a history of cerebrovascular disease (CVD) including ischemic or hemorrhagic strokes were based on patient self-reporting and clinical records. Aneurysm-specific factors, such as aneurysm size, location, and multiplicity, were analyzed by computed tomography angiography or magnetic resonance angiography. Patients were excluded based on any of the following criteria: dyed or curled hair, under 18 years of age, steroid treatment within the past 12 months, or diagnosis of Cushing's disease.

### 2.2 Hair collection, preparation, and cortisol measurement

**Figure 1:**
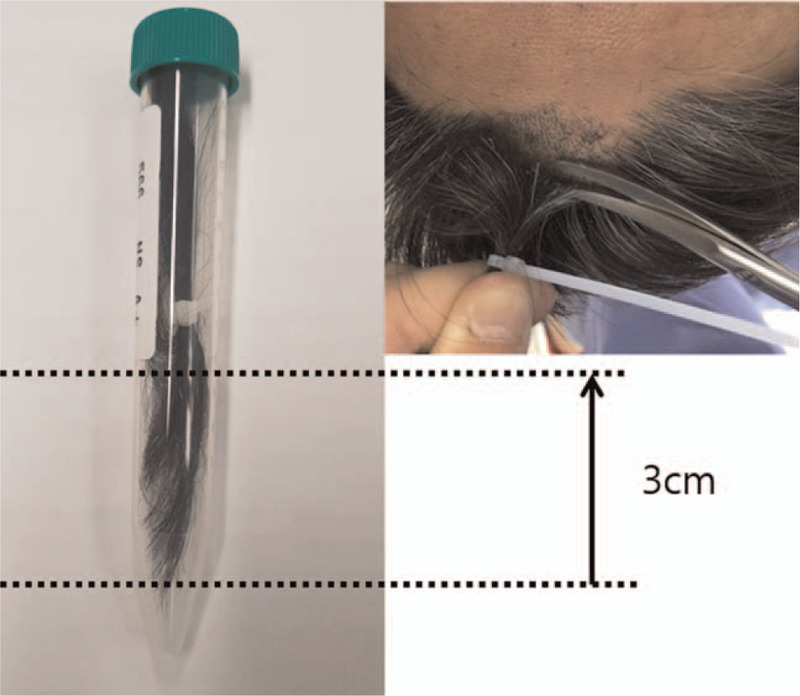
Approximately 50 hair strands were cut from the anterior vertex with sterile scissors as close to the scalp as possible. The proximal 3 cm of hair, reflecting roughly 3 months of cortisol exposure before sampling, was used for the measurement of cortisol concentrations.

Scalp hair was obtained from the unruptured group in the outpatient clinic and from the ruptured group in the operating room at the point of rupture. Approximately 50 strands of hair were cut from the anterior vertex with sterile scissors as close to the scalp as possible. The proximal 3 cm of hair, reflecting roughly 3 months of cortisol exposure before sampling, was used for cortisol measurement (Fig. 1). The hair sample was prepared according to a standardized protocol that has been described in detail.^[22]^ The collected hair was washed twice with isopropanolol to remove any contamination from sweat, sebum, or topical steroids. A minimum of 10 mg of hair (range, 10.0 mg to 30.8 mg) was weighed and ground into small pieces using a bead beater. Methanol was then added to the tubes, and cortisol was extracted overnight using a hair rotator. Methanol was evaporated under 40^°^C nitrogen gas until samples were completely dry. Cortisol concentrations in hair extracts were then measured using a commercial ELISA kit for salivary cortisol (ALPCO, Salem, NH USA). Cross-reactivity (specificity) of the kit with other steroids was specified as follows: prednisolone (13.6%), corticosterone (7.6%), deoxycorticosterone (7.2%), progesterone (7.2%), and cortisone (6.2%).

The sensitivity of the kit was 1.0 ng/mL. Intra-assay and inter-assay coefficients of variation were <10% as reported by the manufacturer.

### 2.3 Statistical analyses

All statistical analyses were performed using IBM SPSS Statistics software (version 18.0; IBM Corp., Armonk, NY USA). To assess the effects of risk factors on aneurysmal rupture, Student's *t*-tests were performed with age, BMI, and hair cortisol concentration. Chi-squared tests were also conducted to determine the differences in sex, aneurysm size, aneurysm location, multiplicity, HTN, type 2 DM, dyslipidemia, smoking status, family history, previous CAD, and previous CVD between the groups.

Data were analyzed separately for males and females considering the fact that there are significant differences in the hair cortisol concentration between the sexes. To determine the impact of sex on hair cortisol concentration, a Student's *t*-test was conducted to determine the differences of hair cortisol concentration between males and females, and then a covariate analysis with sex adjustment was performed. To determine the correlation between the factors that were shown to be statistically different between the two groups and the hair cortisol concentration, additional Student's *t*-tests were performed.

Finally, a logistic regression analysis with backward elimination was used to determine independent risk factors. All *P*-values less than .05 indicated statistically significant differences.

## 3 Results

Baseline characteristics of the two groups, including patient-specific factors and aneurysm-specific factors as well as hair cortisol concentrations are summarized in Table 1.

**Table 1:** Baseline characteristics of the ruptured and unruptured groups.

	Ruptured group (n = 30)	Unruptured group (n = 38)	OR (95% CI)	*P*-value
Age (years)	55.2 ± 15.2	55.9 ± 9.8		.823
Sex, n (%)			1.172 (0.389–3.527)	.786
Male	22 (73.3%)	29 (76.3%)		
Female	8 (26.7%)	9 (23.7%)		
Aneurysm size, n (%)			4.714 (1.525–15.543)	.044^∗^
< 5.0 mm	8 (26.7%)	24 (63.2%)		
> 5.0 mm	22 (73.3%)	14 (36.8%)		
Aneurysm location, n (%)			2.125 (0.541–8.353)	.318
Anterior circulation	24 (80.0%)	34 (89.5%)		
Posterior circulation	6 (20.0%)	4 (10.5%)		
Aneurysm multiplicity, n (%)			1.611 (0.365–7.452)	.075
Single	20 (66.7%)	29 (76.3%)		
Multiple	10 (23.3%)	9 (23.7%)		
BMI (kg/m2)	22.9 ± 3.6	24.0 ± 3.4		.201
HTN, n (%)	10 (33.3%)	22 (57.9%)	0.364 (0.134–0.984)	.044^∗^
Family history, n (%)	7 (23.3%)	5 (13.2%)	2.001 (0.587–2.547)	.412
Type 2 DM, n (%)	8 (26.7%)	6 (15.8%)	1.795 (0.369–8.719)	.691
Dyslipidemia, n (%)	7 (23.3%)	8 (21.1%)	0.833 (0.130–5.337)	>.99
Current smoker, n (%)	9 (30.0%)	8 (13.2%)	2.009 (0.567–7.118)	.344
Previous CAD, n (%)	5 (16.7%)	7 (18.4%)	0.833 (0.130–5.337)	>.99
Previous CVD, n (%)	12 (40%)	6 (15.8%)	4.400 (1.337–14.476)	.022^∗^
Hair cortisol (ng/dL)	55.8 ± 22.0	19.1 ± 6.4		<.001^∗^

**Figure 2:**
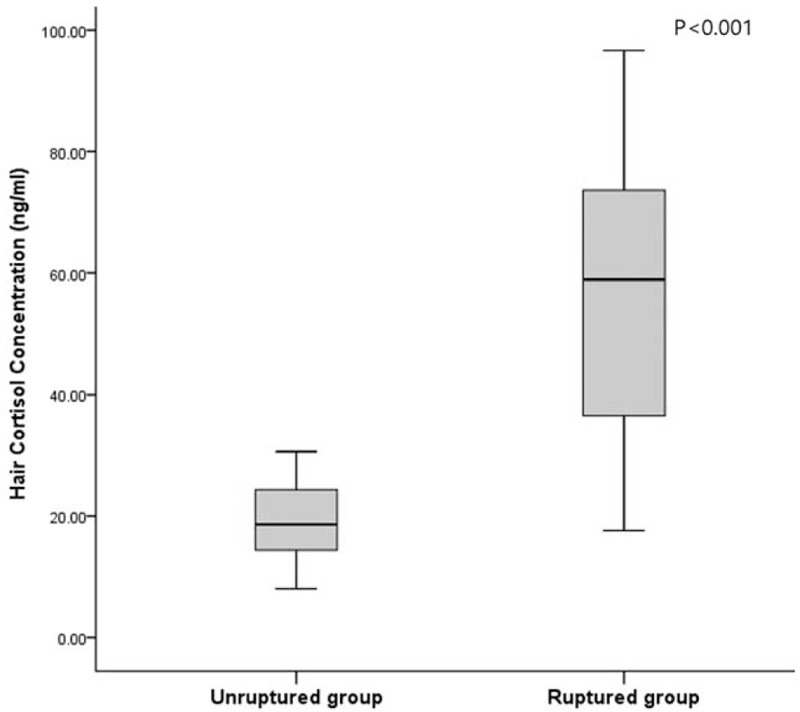
The distribution of mean (± standard error) hair cortisol concentration in the ruptured group and the unruptured group.

**Figure 3:**
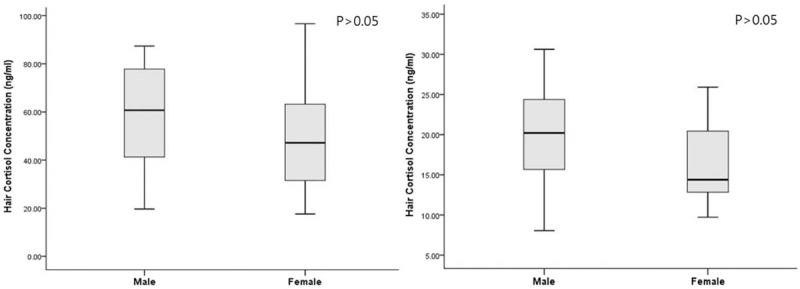
The distribution of mean (± standard error) hair cortisol concentration between the sexes in the ruptured group (left) and the unruptured group (right).

The patients consisted of 51 males (75.0%) and 17 females (25.0%) with a mean age of 54 (range, 30–82 years). Among patient-specific factors, age, sex, BMI, smoking status, prevalence of type 2 DM, dyslipidemia, and prior CAD were not significantly different between the two groups. The prevalence of HTN was significantly higher in the unruptured group than in the ruptured group (*P* = .044). The prevalence of prior CVD was significantly higher in the ruptured group than in the unruptured group (*P* = .022). Regarding aneurysm-specific factors, the mean aneurysm size was 5.6 millimeters, and most of them were located in anterior circulation (n = 58, 85.3%). Aneurysm size was larger in the ruptured group than in the unruptured group (6.0 ± 2.9 vs. 4.8 ± 1.6; p = 0.044), however, aneurysm location and aneurysm multiplicity were not significantly different between the two groups. Hair cortisol concentration was higher in the ruptured group than in the unruptured group (Fig. 2; 55.8 ± 22.0 ng/dL vs. 19.1 ± 6.4 ng/dL; *P* < .001). To evaluate the effect of sex on hair cortisol concentration, data were analyzed separately for males and females. The overall mean hair cortisol concentration of males was 36. 4 ± 23.8 ng/dL and was not significantly different from the level among females (32.1 ± 24.3 ng/dL). Further, no significant difference was detected in hair cortisol concentration between males and females in both the ruptured and unruptured groups (Fig. 3). Finally, hair cortisol concentration was significantly different between the two groups on covariance analysis with sex adjustment (Table 2; 55.9 ± 2.8 ng/dL vs. 19.1 ± 2.5 ng/dL; *P* < .001).

**Table 2.** Sex-adjusted mean of hair cortisol concentration between ruptured group and unruptured group.

	Ruptured group	Unruptured group	*P*-value
Sex, n (%)			.786
Male	22 (73.3%)	29 (76.3%)	
Female	8 (26.7%)	9 (23.7%)	
Hair cortisol (ng/ml)	55.9 ± 2.8	19.1 ± 2.5	<.001^∗^

In the logistic regression analysis between the two groups, hair cortisol concentration was significantly associated with aneurysmal rupture (odds ratio [OR]: 2.245, 95% confidence interval [CI]: 1.825–2.753; *P* = .013).

Previous CVD was another independent risk factor for aneurysmal rupture (OR: 1.577, 95% CI: 1.099–2.262; *P* = .040). However, HTN and aneurysm size were not associated with aneurysmal rupture in this analysis (Table 3).

**Table 3.** Multivariate analysis of risk factors for aneurysmal rupture.

Variables	B	*P*-value	OR (95% CI)
Smoking	0.109	.130	1.056(.952–2.125)
HTN	−3.931	.074	0.020 (0.000–1.465)
Aneurysm size	0.489	.445	1.624 (1.062–2.482)
Previous CVD	0.455	.040^∗^	1.577 (1.099–2.262)
Hair cortisol (ng/dL)	0.843	.013^∗^	2.245 (1.825–2.753)

## 4 Discussion

To identify additional risk factors for aneurysmal rupture, we investigated the association between aneurysmal rupture and chronic stress as measured by hair cortisol concentration.

We found that hair cortisol concentration was higher in the ruptured group than in the unruptured group and that patients with higher hair cortisol concentrations were 2.25 times more prone to rupture than those with lower hair cortisol concentrations. This suggests that high long-term cortisol concentration might be an independent risk factor for aneurysmal rupture.

There are some possible biological mechanisms to explain how high long-term hair cortisol concentration affects aneurysmal rupture.

Before explaining mechanisms, we need to understand the terms “allostasis” and “allostatic load.” Broadly, stress is defined as many events of daily life promoting activities of the physiological systems causing some wear and tear. This proves to be essential for adaptation to the environment causing alterations in hemostasis. Allostasis means “maintaining hemostasis through change” and wear and tear is called the “allostatic load.” Allostasis and allostatic load begin with primary stress mediators such as cortisol and catecholamines. Stress and allostatic load in the short-term protect the body and promote adaptation, however, in the long-term they cause changes in the body leading to chronic diseases.^[11,23]^

Explained in other words, when stress stimuli are chronically maintained, circulating cortisol remains at high levels over a prolonged period, resulting in a higher accumulation of cortisol in hair. This affects the immune system and activates the inflammatory pathway leading to vascular wall injury.^[23,24]^ In addition, high long-term cortisol causes a failure to shut off the surge in blood pressure that contributes to vascular endothelial dysfunction.^[25]^

Additionally, patients who suffered from ischemic or hemorrhagic strokes in the past had a 1.58 increase in the odds of aneurysmal rupture (*P* = .040). Ishikawa et al. showed that incidental aneurysms were found in 3.5% of patients with ischemic stroke and in 4.7% of patients with hemorrhagic stroke.^[26]^ Another study showed that cerebral aneurysms were found in 6.1% of patients with ischemic stroke, which is higher than that in the general population.^[27]^ The increased risk of aneurysmal rupture in patients who have suffered a stroke may be explained by the fact that these patients have higher rates of incidental aneurysms and share common risk factors. Patients who have suffered a stroke and are taking antithrombotic drugs and those with higher rates of hypertension may have a significantly higher risk of bleeding or aneurysmal rupture.

Unexpectedly, we did not find any significant correlations between aneurysmal rupture and other well-established risk factors among both patient-specific factors (HTN, family history, DM, and smoking status) and aneurysm-specific factors (aneurysm size, location, and multiplicity).

In addition, some of the known risk factors (smoking, HTN, aneurysm size, and previous CVD) did not correlate with the hair cortisol concentration. These results may be due to the small number of patients used in our study; however, these risk factors have exhibited inconsistent relationships with aneurysmal rupture in previous studies.^[2,28]^

Several limitations of our study need to be discussed. First, although prospective, our study was a single-center study with a small sample size of only 68 patients. Moreover, the prevalence of underlying chronic diseases was low, which may have resulted in limited statistical significance. Second, hair cortisol analysis was performed using a salivary cortisol kit. This may have overestimated hair cortisol concentrations due to its cross-reactivity.

Third, it is somewhat clear that males and females respond differently to stress; however, we did not find sex differences in the hair cortisol concentration. This may be due to our small sample size, especially the number of females (17 out of 68 patients). However, many studies have reported no sex differences in the hair cortisol concentration.^[22,29,30]^ Therefore, further research is needed to identify differences in hair cortisol concentration between the sexes with more detailed mechanisms.

Finally, we did not investigate the relationship between hair cortisol concentration and the perceived chronic stress, which is measured by self-reporting and questionnaires. However, O’Brien et al suggested that hair cortisol concentration may not always correlate with a single stress index, but may correlate with global assessment of chronic stress.^[18]^

Though this study has several limitations, our finding of a correlation between aneurysmal rupture and hair cortisol concentration could present a new direction for identifying valuable risk factors. It would be desirable to include a large sample size and an assessment of the association with perceived stress in future studies. Moreover, in addition to aneurysm, it is expected that future studies could be extended to other brain diseases including ischemic strokes.

## 5 Conclusion

This is the first study to find an association between aneurysmal rupture and chronic stress as measured by hair cortisol concentration. We found a 2.25 increase in the odds of aneurysmal rupture in higher hair cortisol concentration. This suggests that high hair cortisol concentration may be an independent risk factor for aneurysmal rupture.

## Author contributions

**Conceptualization:** Tae-Sun Kim.

**Data curation:** You-Sub Kim, Dong-Jun Song, Tae-kyu Lee.

**Formal analysis:** You-Sub Kim.

**Investigation:** Dong-Jun Song.

**Writing – original draft:** You-Sub Kim, Sung-Pil Joo.

**Writing – review & editing:** You-Sub Kim.
